# Human Leukocyte Antigen Class II Alleles (DQB1 and DRB1) as Predictors for Response to Interferon Therapy in HCV Genotype 4

**DOI:** 10.1155/2013/392746

**Published:** 2013-03-14

**Authors:** Olfat Shaker, Heba Bassiony, Maissa El Raziky, Samer S. El-Kamary, Gamal Esmat, Akmal M. El-Ghor, Mona M. Mohamed

**Affiliations:** ^1^Departments of Medical Biochemistry & Molecular Biology, Faculty of Medicine, Cairo University, Cairo, Egypt; ^2^Department of Zoology, Faculty of Science, Cairo University, Cairo 12613, Egypt; ^3^Departments of Tropical Medicine & Hepatology, Faculty of Medicine, Cairo University, Cairo, Egypt; ^4^Department of Epidemiology and Public Health, University of Maryland School of Medicine, Baltimore, MD, USA

## Abstract

Human leukocyte antigens class II play an important role in immune response against HCV. We investigated whether HLA class II alleles influence susceptibility to HCV infection and response to interferon therapy. HLA-DRB1 and -DQB1 loci were genotyped using PCR-SSO Luminex technology. According to our regimen, 41 (66%) of patients achieved sustained virological response to combined treatment of IFN and ribavirin. Frequencies of DQB1∗0313 allele and DRB1∗04-DRB1∗11, DQB1∗0204-DQB1∗0313, DQB1∗0309-DQB1∗0313, and DQB1∗0313-DQB1∗0319 haplotypes were significantly more frequent in nonresponders than in responders. In contrast, DQB1∗02, DQB1∗06, DRB1∗13, and DRB1∗15 alleles were significantly more frequent in responders than in nonresponders. Similarly, DRB1∗1301, DRB1∗1361, and DRB1∗1369 alleles and DRB1∗1301-DRB1∗1328, DRB1∗1301-DRB1∗1361, DRB1∗1301-DRB1∗1369, DRB1∗1328-DRB1∗1361, and DRB1∗1328-DRB1∗1369 haplotypes were significantly found only in responders. Some alleles and linkages showed significantly different distributions between patient and healthy groups. These alleles may be used as predictors for response to treatment or to susceptibility to HCV infection in the Egyptian population.

## 1. Introduction 

Hepatitis C virus is a major public health problem worldwide; more than 130–170 million people are infected. Egypt has approximately 13% (about 10 millions) anti-HCV positive individuals mainly genotype 4a [1, 2]. Most of them are chronically infected with risk to develop liver cirrhosis or hepatocellular carcinoma (HCC) [3]. Combination therapy of pegylated interferon and ribavirin has been recommended and approved for patients with HCV infection [4]. But treatment is costly and causes many side effects [5]. Also not all patients who receive antiviral therapy are able to clear the virus and respond to treatment; only about 55% of patients can successfully clear the virus depending on virological factors and host factors including immunogenetic factors [6, 7]. 

The host immune response is initiated by presentation of viral peptides in the context of human leukocyte antigen (HLA class II) molecules on the surface of antigen presenting cells to CD4^+^T cells [8]. HLA is an extended region on the short arm of human chromosome six between 6p21.31 and 3p21.32.1; and is divided into three subregions: HLA class I, class II, and class III [9]. It is composed of six major genes A, B, C, DR, DQ, and DP. The first three genes of class I influence presentation of endogenous antigens, while the other three genes of class II influence presentation of exogenous antigens so it affects the outcome of various infectious diseases [10]. 

 HLA alleles are highly polymorphic among different populations which give variation in immune response [11]. Several studies in different populations demonstrated the association between HLA alleles and the outcome of HCV infection, but associated alleles are highly variable from one population to another [12, 13]. It was reported that HLA class II alleles, particularly DRB1 and DQB1 alleles, play a critical role in the outcome of HCV infection, influence susceptibility to or protection from HCV infection, and also affect response to antiviral treatment [14–16].

 A Brazilian study found that DRB1*11 and/or DQB1*03 alleles might be responsible for selection of viral epitopes for presentation to CD4^+^T cells, leading to an efficient immune response against the virus [17]. In Pakistani patients, HLA-DRB1*11 and -DQB1*0301 alleles are associated with viral clearance, but HLA-DRB1*07 individually or in combination with HLA-DQB1*02 was associated with viral persistence [18]. However, Chinese patients with DRB1*07 allele presented almost complete response to treatment, while DRB1*04 is associated with no responsiveness [19]. Another study showed that HLA-DQB1*0301 allele is associated with SVR; also there is a synergism between the HLA-DQB1*0301 and HLA-A*0201 alleles increasing the SVR rate [20]. Finally in a recent Egyptian study, HLA DRB1*01, DQB1*03, and DQB1*05 alleles were associated with viral clearance, while DRB1*7 and DQB1*02 alleles were associated with viral persistence [21].

## 2. Aim of Work

 We conducted the present study to assess the association between HLA class II (DRB1 and DQB1) alleles and response to combined interferon *α*-2b/ribavirin therapy in HCV infected Egyptian patients and to investigate whether these alleles influence the susceptibility to or protection from HCV infection. 

## 3. Materials and Methods

### 3.1. Study Population

Sixty-two HCV chronically infected Egyptian patients (51 males, 11 females; mean age 40.53 ± 1.16 years; range 20–59) were enrolled in our study after signing consent forms and were followed from September 2009 to September 2011 at outpatient clinics of Tropical Medicine and Hepatology Departments, El-Kasr El-Aini Hospital, Cairo University. The study protocol and informed consent were approved by the Ethical Committee of Cairo University. All patients were positive for HCV antibodies and they had detectable HCV-RNA with genotype 4 and elevated aminotransferases, liver biopsy showing histological evidence of chronic hepatitis, and they were never previously treated with interferon. Patients were negative for hepatitis B surface antigen (HBsAg), autoimmune hepatitis, antinuclear antibodies, human immunodeficiency virus (HIV), and active schistosomiasis. Fifty-seven healthy volunteers were included as control group (32 males, 25 females; mean age 34.63 ± 1.997 years; range 22–50).

### 3.2. Study Design

Patients received subcutaneous injections of either pegylated interferon *α*-2b (100 *µ*g/week) or standard interferon *α*-2b (3 million units three times/week) combined with orally given ribavirin (1000–1200 mg/day, body weight based). All patients were followed for 72 weeks. HCV-RNA was tested at weeks 24, 48, and 72; patients who were tested negative with undetectable HCV-RNA were classified as responders. While those tested positive with detectable HCV-RNA were classified as nonresponders. 

Standard baseline laboratory tests included liver function tests of aminotransferases, alkaline phosphatase, direct and total bilirubin, albumin and prothrombin time and concentration, complete blood picture (CBC), antinuclear antibodies, alpha-fetoprotein (AFP), thyroid stimulating hormone (TSH), electrocardiogram, abdominal ultrasound, and liver biopsy examination to estimate the grade of activity and fibrosis according to the Metavir scoring system as follows: for activity, A0 is no histological activity, A1 mild activity, A2 moderate activity, and A3 severe activity; for fibrosis, F0 is no fibrosis, F1 portal tract expansion by fibrosis, F2 less than 50% bridging fibrosis, F3 more than 50% bridging fibrosis without cirrhosis, and F4 established cirrhosis [22].

### 3.3. HLA Class II Genotyping

Genomic DNA was extracted and purified from leukocytes of peripheral blood samples using QIAamp DNA minikit (Qiagen Inc., Valencia, CA, USA), according to the manufacturer's instructions. DNA was resuspended in 200 *µ*L elution buffer and stored at −80°C to be used for PCR. HLA class II genotyping was performed at allele level for DRB1 and DQB1 regions using LABType SSO Typing Test, which applies Luminex technology as recommended by Luminex Corporation. We used two kits which are LAPType SSO DRB1 and LAPType SSO DQB1 (One Lambda Inc., USA). First, target DNA was amplified using specific primer sets that are biotinylated at 5 terminus for labeling the PCR product to allow its detection by R-Phycoerythrin-conjugated Streptavidine (SAPE). PCR product was denatured, neutralized, and rehybridized to oligonucleotide probes bound to fluorescently coded microbeads. Then biotinylated hybridized amplicons on microbeads were allowed to be labeled with SAPE. Luminex 100 flow analyzer detected the fluorescent intensities of Phycoerythrin (PE) on each microbeads. Luminex 100 flow analyzer identified HLA alleles via HLA visual 1.0 software by referring to HLA typing template data for DRB1 and DQB1 provided by kits manufacturer (One Lambda Inc.).

### 3.4. Statistical Analysis

Our data were analyzed using SPSS version 18.0. Chi square (*χ* ^2^) test or Fisher's exact test was used to compare alleles' frequencies, gender, viral load, grade of activity, and fibrosis between groups, while the student's *t*-test was used to compare age, liver enzymes, and *α*-fetoprotein. *P*  values ≤ 0.05 is considered statistically significant. The Bonferroni correction was applied to the *P*-values in order to avoid a type I error in the analysis for 39 DQB1 alleles and 140 DRB1 alleles. We used continuity correction asymptotic 2-tail significance if the number of samples was less than sixty. Classification and Regression Trees (CART) were used to classify the patients' predictors for infection and response to treatment included age, HCV level, and the studied alleles.

## 4. Results

### 4.1. Patients' Clinical, Virological, and Histological Features

Table 1 shows the baseline features of sixty-two patients; we compared related factors affecting response to treatment in responder and nonresponder patients to treatment. Age, ALT, AST, and AFP are represented by mean ± standard error of mean. According to response to treatment, 41 (66.1%) patients were responders and 21 (33.9%) were nonresponders to combination therapy of interferon *α*-2b and ribavirin. Patients with lower ALT, HCV RNA level, liver activity, and fibrosis tend to be responder to treatment with statistically significant difference (*P* ≤ 0.05). 

### 4.2. Gel Electrophoresis of PCR Product for Specific Locus of DNA Samples

**Figure 1:**
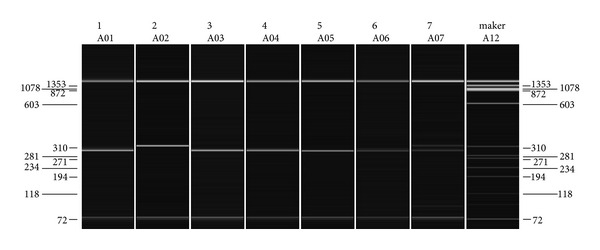
Image of gel electrophoresis representative to separation of seven samples of PCR product for amplified DRB1 locus. Lanes from 1 to 7 contain DNA samples; the last lane contains QX DNA size marker (ΦX174/HaeIII) with fragments of 72, 118, 194, 234, 271, 281, 310, 603, 872, 1078, and 1353 bp. Upper and lower bands are for the alignments, and in between is the band of successful PCR product. QIAxcel device with BioCalculator Analysis Software (Qiagen Inc., Valencia, CA, USA).

Figure 1 shows an image for separation of amplified PCR product of DRB1 locus by agarose gel electrophoresis using QIAxcel device with BioCalculator Analysis Software (Qiagen Inc., Valencia, CA, USA). It is a representative to seven samples with QX DNA size marker. The upper and lower bands are the alignments which were added to each sample. 

### 4.3. Comparison of HLA-DQB1 Alleles among Groups

As shown in Table 2, the frequencies of 39 DQB1 alleles were compared between patients and healthy group and also between responders and nonresponders. We collapsed four digit alleles into their respective two digit categories (familial alleles). For instance, DQB1*0201, DQB1*0202, DQB1*0203, DQB1*0204, and DQB1*0205 were grouped together as DQB1*02.

It was observed that DQB1*0201, DQB1*0202, DQB1*0204, DQB1*0301, DQB1*0309, DQB1*0319, and DQB1*06 alleles were significantly more frequent in patients with HCV than in healthy controls. On the other hand, DQB1*0205, DQB1*0303, DQB1*0312, DQB1*0315, DQB1*0320, and DQB1*03 alleles were significantly more frequent in healthy controls than in patients.

Meanwhile, the only DQB1*0313 allele was significantly more frequent in nonresponders (25%) than in responders (5.3%). DQB1*02 and DQB1*06 were found to be significantly higher in responders (63.2%, 44.7%) than in nonresponders (30%, 10%) (Table 2).

### 4.4. Comparison of HLA-DRB1 Alleles among Groups

The distribution of 140 HLA-DRB1 alleles was compared in responders and nonresponders; DRB1*1301, DRB1*1361, and DRB1*1369 alleles were significantly found only in responders (17.1% for each) and are absent in nonresponders (0%). DRB1*13 and DRB1*15 were significantly more frequent in responders (46.3%, 24.4%) than, in nonresponders (14.3%, 4.8%) (Table 3).

DRB1*07, DRB1*0701, DRB1*0703, DRB1*0705, DRB1*0706, DRB1*0707, DRB1*0708, DRB1*0709, DRB1*0710N, DRB1*0711, DRB1*0712, DRB1*0713, and DRB1*0714 alleles were significantly more frequent in healthy individuals than in patients, while DRB1*13 was significantly more frequent in infected patients than in controls (Table 3).

### 4.5. Comparison of Linked Familial Alleles (Haplotypes) by Their First Two Digits between Groups

The frequencies of 70 linkages with their first two digits were compared between groups. It was observed that the frequency of DRB1*04-DRB1*11 haplotype was significantly higher in nonresponders (14.3%) than in responders (0%). Frequencies of DQB1*02-DRB1*07, DQB1*03-DRB1*07, and DQB1*02-DQB1*03 haplotypes were significantly higher in healthy controls than in HCV patients (Table 4).

Regardless of HCV infection, we noticed that HLA-DQB1*02, DQB1*03, DRB1*07, and DRB1*13 were more frequent in the studied candidates of the Egyptian population.

### 4.6. Comparison of Linked Alleles (by Their Four Digits) between Groups

 When linked haplotypes of DQB1 with DQB1 of both responders and nonresponders and DRB1 with DRB1 of both groups also; DQB1*0204-DQB1*0313, DQB1*0309-DQB1*0313, and DQB1*0313-DQB1*0319 were statistically significant in nonresponders. While DRB1*1301-DRB1*1328, DRB1*1301-DRB1*1361, DRB1*1301-DRB1*1369, DRB1*1328-DRB1*1361, and DRB1*1328-DRB1*1369 were statistically significant in responders (Table 5). 

## 5. Discussion

A number of baseline factors were reported as independent predictors for the response to interferon therapy including, low viral load, HCV genotype other than genotype 1b, absence of liver cirrhosis, low steatosis, absence of coinfection [23, 24], and also host immunogenetic factors including human leukocyte antigen (HLA) genes [25, 26]. Several studies have shown that high viral load and high grade of liver activity and fibrosis are negatively correlated with sustained virological response to interferon treatment [27, 28]. We agree with these studies as we noticed that responders to treatment are significantly characterized by lower ALT, lower viral load, and lower grade of liver activity and fibrosis. 

Genetic factors of the patient strongly influence the outcome of viral infection and the response of CD4^+^T cells. Polymorphism in the peptide binding regions of HLA-I and HLA-II molecules determines antigenic specificities and strength of immune response to a given pathogen. It is well known that HLA class II genes are crucial factors regulating cellular and humoral immune response through producing HLA class II molecules participating in presentation of viral antigens on the surface of antigen presenting cells to CD4^+^T cells [29, 30]. Genes of DR and DQ, which are of the major loci on HLA class II region, play an important role in disease progression and protection from HCV infection and are prominent immunogenetic factors influencing response to interferon treatment [16]. Presence of specific HLA alleles or haplotypes may affect the response to IFN therapy in HCV infection [31, 32], but associations vary among populations and results are controversial. In this regard HLA study may help to identify response and no response markers that help to select suitable patients who are the most likely to benefit from IFN therapy. Also identification of protective HLA alleles may provide clues to development of vaccine.

The present work investigated polymorphism in HLA class II (DRB1 and DQB1) genes in the Egyptian patients with HCV infection who received combined treatment of interferon and ribavirin. PCR-SSO Luminex method was used that provides high resolution of typing to identify the specific alleles (by their four digits) of DRB1 and DQB1 loci. Luminex technology was previously used for HLA typing by Dai et al. [7], Itoh et al. [33], and by different studies [7, 33, 34]. To our knowledge, this study is the first report to test whether there is an association between HLA alleles and response to HCV treatment in the Egyptian population.

 Interestingly, our results detected a statistically significant difference in the distribution of HLA-DRB1 and -DQB1 alleles and their haplotypes between responders and nonresponders. The frequencies of DQB1*0313 allele and DRB1*04-DRB1*11, DQB1*0204-DQB1*0313, DQB1*0309-DQB1*0313, and DQB1*0313-DQB1*0319 haplotype were significantly higher in nonresponders than in responders. On the other hand, DQB1*02, DQB1*06, DRB1*1301, DRB1*1361, DRB1*1369, DRB1*13, and DRB1*15 and DRB1*1301-DRB1*1328, DRB1*1301-DRB1*1361, DRB1*1301-DRB1*1369, DRB1*1328-DRB1*1361, and DRB1*1328-DRB1*1369 alleles were significantly more frequent in responders than in nonresponders. 

 Similarly, other studies in different populations reported that response or no response to treatment was correlated to presence of specific HLA alleles or significant haplotype linkages. As in Taiwan, HLA-DRB1*15 allele was positively correlated with a sustained response to IFN-*α* [25]. Also we agree with a recent study conducted in Egypt on 56 patients from Menoufiya [21] that DQB1*03 and DQB1*05 are associated with viral clearance, and we found both alleles are significantly more frequent in responders than in nonresponders. But we disagree with them in that DRB1*01 is associated with viral clearance and DRB1*7, DQB1*02 alleles are associated with viral persistence. This difference may be due to different population or treatment regimens or the type of interferon. In a recent cohort study on Pakistani HCV patients on IFN therapy, HLA-DRB1*11 and DQB1*0301 were found to be associated with viral clearance. While, HLA-DRB1*07 individually or in combination with HLA-DQB1*02 was found to be associated with viral persistence [18]. An Egyptian study of hemophilic children and adults showed that certain HLA-DR alleles as DRB1*0101 and DRB1*0301 may have role in HCV clearance and persistence [35].

In contrast to our study, in the Polish population, DRB1 alleles were not associated with response to IFN treatment, although DRB1*07 and DRB1*13 were more frequent in nonresponders and responders to treatment, respectively, but not statistically significant [36]. 

In France, DQB1*06 was associated with responsiveness to interferon therapy in patients with HCV genotype 1 [37]. In Caucasian patients from Canada, response to treatment was associated with DRB1*0404 allele [30], but with DRB1*07 allele in Chinese patients [19]. In Taiwan, HLA-DRB1*15, HLA-A11, HLA-B51, and HLA-Cw15 alleles were positively correlated with a sustained response, whereas HLA-A24 allele was inversely associated with response to IFN-*α* [32], while another study in Taiwan found that B40-DRB1*3, B46-DRB1*9, Cw1-DQB1*3, Cw1-DRB1*9, and DQB1*3-DRB1*9 haplotypes were associated with sustained virological response to treatment [7]. In Japan, DRB1*0404 was associated with no response to treatment, while HLA-B54 and HLA-A24-B54-DR4 haplotypes should be predictors for poor response to IFN therapy in patients with chronic hepatitis C [31]. In Poland DRB1*0701-DQA1*0201-DQB1*02 haplotype was associated with response to IFN-*α* [36]. Study on patients from the United States observed that the A*02, B*58, and DPB1*1701 HLA alleles were independently associated with SVR even after adjustment for other predictors of response such as race, gender, baseline viral load, severity of liver fibrosis, and dosage of medication taken [38].

Meanwhile, other studies found no difference in distribution of HLA alleles between responders and nonresponders to antiviral therapy, for instance, in France [39, 40], in Italy [41], in Europe [42], and in a Brazilian study [43, 44]. In German patients suffering from chronic hepatitis C and treated with IFN-*α*, it was found that pretreatment viral factors, not host factors, were significantly correlated with treatment response [45], In Spanish population, although HLA class II showed no effect on response to interferon treatment, HLA class I-B44 was associated with response to combined therapy of IFN and ribavirin, not with interferon alone [46].

Comparing the distribution of HLA-DRB1 and -DQB1 alleles and their haplotypes between HCV patients and healthy group revealed that DQB1*0201, DQB1*0202, DQB1*0204, DQB1*0301, DQB1*0309, DQB1*0319, DQB1*06, and DRB1*13 alleles were significantly more frequent in patients than in healthy group. On the other hand, DQB1*0205, DQB1*0303, DQB1*0312, DQB1*0315, DQB1*0320, DRB1*0701, DRB1*0703, DRB1*0705, DRB1*0706, DRB1*0707, DRB1*0708, DRB1*0709, DRB1*0710N, DRB1*0711, DRB1*0712, DRB1*0713, DRB1*0714, DQB1*03, and DRB1*07 alleles and DQB1*02-DRB1*07, DQB1*03-DRB1*07, and DQB1*02-DQB1*03 haplotypes were more frequent in healthy controls than in patients. Those alleles may enhance the immune response against HCV infection.

 Similarly a German study of North Europeans found that DRB1*13 was associated with susceptibility to HCV infection [47]. Also in Thailand, DRB1*0701 was significantly higher in uninfected controls compared with HCV infected patients [48]. Studies conducted in France and Turkey reported the protective effect of DRB1*11 in healthy controls [14, 49]. In contrast our results showed that DRB1*11 was more frequent in healthy group than in patients, but difference was not statistically significant. Our study demonstrated that presence of group of suballeles of DQB1*03 was protective against HCV infection, not only presence of DQB1*0301 individual allele as reported by several studies.

 However, in another Egyptian study, DQB1*06 was significantly more frequent in healthy controls than in patients with HCC [50]. A Brazilian study reported that DRB1*07 allele was associated with chronic infection with HCV and was more frequent in patients than in healthy controls [43, 44]. Also in Poland, DRB1*0701 was frequently higher in patients than in controls [51]. In Korean, DQB1*0604 was significantly higher in patients with HCV infection than in healthy controls, and DQB1*0201 and DQB1*0301 were protective alleles found more frequent in uninfected control than in patients [52]. 

In conclusion, the present study suggested that certain HLA-DRB1, -DQB1 alleles, DQB1*02, DQB1*06, DRB1*13, DRB1*15, DRB1*1301, DRB1*1361, and DRB1*1369, may act as predictors for response to treatment in the Egyptian population. This is important for disease management and deciding which patient would most likely benefit from IFN therapy and which would not. It is strongly recommended that further investigations are needed for more genetic factors (non-HLA genes) and association of these factors with HLA type to affect response to interferon treatment in the Egyptian population which may help selecting HCV patients suitable for antiviral therapy.

## Figures and Tables

**Table 1:** Baseline clinical, virological, and histological features of sixty-two HCV patients and comparison of these features between responder and nonresponder patients to treatment.

Characteristics	HCV patients	Responders	Nonresponders	*P* value
	*N* = 62	*N* = 41 (66.1%)	*N* = 21 (33.9%)	
Male/female	51/11	34/7	17/4	1.000
Age (mean ± S.E)	40.53 ± 1.17	39.44 ± 1.5	42.67 ± 1.67	0.160
Liver function				
ALT (IU/L)				
(mean ± S.E)	73.9 ± 4.5	67.93 **± **5	85.62 ± 8	**0.041** ^ #^
AST (IU/L)				
(mean ± S.E)	61.6 ± 4.9	61.44 **± **7.13	61.9 ± 4.5	0.956
AFP (U/L)				
(mean ± S.E)	7.1 ± 1.5	6.7 **± **2.1	7.78 **± **1.6	0.692
HCV-RNA level				
Low	20 (32.3%)	12 (29.3%)	8 (38.1%)	**0.035** ^ #^
Moderate	27 (43.5%)	21 (51.2%)	6 (28.6%)	
High	5 (8.1%)	2 (4.9%)	3 (14.3%)	
Unknown	10 (16.1%)	6 (14.6%)	6 (28.6%)	
Activity score				
A1	49 (79%)	35 (85.4%)	14 (66.7%)	**0.044** ^ #^
A2	13 (21%)	6 (14.6%)	7 (33.3%)	
Fibrosis score				
F1	28 (45.2%)	21 (51.2%)	7 (33.3%)	**0.05** ^ #^
F2	20 (32.3%)	14 (34.1%)	6 (28.6%)	
F3	14 (22.6%)	6 (14.6%)	8 (38.1%)	

^#^ Significant difference at *P* ≤ 0.05; between responder and nonresponder patients to treatment. No patients had A0 or A3 activity and F0 or F4 fibrosis.

**Table 2:** Frequencies and percentages of DQB1 alleles in HCV patients and healthy controls and in responders and nonresponders to combined treatment of interferon and ribavirin.

DQB1	HCV patients	Healthy controls	PC	Responders	Nonresponders	PC
	(*n* = 62)	(*n* = 57)		*N* = 41	*N* = 21	
*02	32 (51.6%)	41 (71.9%)	0.175	24 (63.2%)	6 (30%)	0.026^#^
*0201	31 (50.0%)	10 (17.5%)	0.015^†^	19 (50%)	10 (50%)	0.609
*0202	30 (48.4%)	10 (17.5%)	0.027^†^	18 (47.4%)	10 (50%)	1.000
*0204	30 (48.4%)	10 (17.5%)	0.027^†^	18 (47.4%)	10 (50%)	0.534
*0205	9 (14.5%)	35 (61.4%)	0.000^†^	7 (18.4%)	1 (5%)	0.241
*03	40 (64.5%)	53 (92.9%)	0.017^†^	27 (71.1%)	11 (55%)	0.255
*0301	27 (43.5%)	7 (12.3%)	0.022^†^	16 (42.1%)	9 (45%)	0.525
*0303	2 (3.2%)	35 (61.4%)	0.000^†^	1 (2.6%)	1 (5%)	0.575
*0309	27 (43.5%)	7 (12.3%)	0.022^†^	16 (42.1%)	9 (45%)	0.525
*0312	2 (3.2%)	35 (61.4%)	0.000^†^	1 (2.6%)	1 (5%)	0.575
*0313	7 (11.3%)	10 (17.5%)	0.695	2 (5.3%)	5 (25%)	0.041^#^
*0315	2 (3.2%)	35 (61.4%)	0.000^†^	1 (2.6%)	1 (5%)	0.575
*0319	27 (43.5%)	7 (12.3%)	0.022^†^	16 (42.1%)	9 (45%)	0.525
*0320	2 (3.2%)	35 (61.4%)	0.000^†^	1 (2.6%)	1 (5%)	0.575
*0321	4 (6.6%)	12 (21.1%)	0.224	3 (7.9%)	1 (5%)	1.000
*05	12 (19.4%)	10 (17.5%)	1.000	8 (21.1%)	3 (15%)	0.428
*0501	32 (51.6%)	41 (71.9%)	0.175	6 (15.8%)	2 (10%)	0.701
*0503	3 (4.8%)	19 (33.3%)	0.166	1 (2.6%)	2 (10%)	0.271
*06	20 (32.3%)	4 (7.0%)	0.030^†^	17 (44.7%)	2 (10%)	0.008^#^
*0601	5 (8.1%)	9 (15.8%)	0.823	3 (7.9%)	2 (10%)	0.568
*0602	2 (3.2%)	15 (26.3%)	0.170	2 (5.3%)	0 (0%)	0.425
*0603	10 (16.1%)	22 (38.6%)	0.171	8 (21.1%)	2 (10%)	0.468
*0627	3 (4.8%)	10 (17.5%)	0.325	1 (2.6%)	2 (10%)	0.271
*0628	10 (16.1%)	7 (12.3%)	0.886	8 (21.1%)	2 (10%)	0.250
*0633	2 (3.2%)	13 (22.8%)	0.173	2 (5.3%)	0 (0%)	0.540
*0634	2 (3.2%)	17 (29.8%)	0.085	1 (2.6%)	1 (5%)	0.575

^#^ Significant difference at PC ≤ 0.05 (corrected *P* after the Bonferroni correction) between responder and non-responder patients to treatment, ^†^Significant difference at PC ≤ 0.05 (corrected *P* after Bonferroni correction) between patients and healthy group. Each patient may have multiple suballeles or none at all, so that totals are not always equal to “*n*.”

**Table 3:** Frequencies and percentages of DRB1 alleles in HCV patients and healthy group and in responder and nonresponder patients to combined treatment of interferon and ribavirin.

DRB1	HCV patients	Healthy controls	PC	Responders	Nonresponders	PC
	(*n* = 62)	(*n* = 57)		(*n* = 41)	(*n* = 21)	
*01	2 (3.2%)	6 (10.5%)	0.215	1 (2.4%)	1 (4.8%)	1.000
*0102	1 (1.6%)	9 (15.8%)	0.148	1 (2.4%)	0 (0.0%)	0.661
*0106	1 (1.6%)	1 (1.8%)	1.000	1 (2.4%)	0 (0.0%)	0.661
*03	13 (21.0%)	25 (43.9%)	0.072	11 (26.8%)	2 (9.5%)	0.187
*0301	8 (12.9%)	17 (29.8%)	0.552	6 (14.6%)	2 (9.5%)	0.447
*0304	4 (6.5%)	12 (21.1%)	0.112	2 (4.9%)	2 (9.5%)	0.417
*0313	5 (8.1%)	0 (0.0%)	0.222	3 (7.3%)	2 (9.5%)	0.556
*0328	8 (12.9%)	0 (0.0%)	0.183	6 (14.6%)	2 (9.5%)	0.447
*0332	3 (4.8%)	0 (0.0%)	0.556	1 (2.4%)	2 (9.5%)	0.263
*04	14 (22.6%)	12 (21.1%)	1.000	11 (26.8%)	3 (14.3%)	0.346
*0403	3 (4.8%)	13 (22.8%)	0.089	2 (4.9%)	1 (4.8%)	0.737
*0471	2 (3.2%)	7 (12.3%)	0.134	2 (4.9%)	0 (0.0%)	0.434
*0445	4 (6.5%)	9 (15.8%)	0.212	2 (4.9%)	2 (9.5%)	0.417
*07	22 (35.5%)	36 (63.2%)	0.029^†^	15 (36.6%)	7 (33.3%)	1.000
*0701	22 (35.5%)	36 (63.2%)	0.029^†^	13 (31.7%)	9 (42.9%)	0.277
*0703	20 (32.3%)	36 (63.2%)	0.013^†^	13 (31.7%)	7 (33.3%)	0.558
*0705	20 (32.3%)	36 (63.2%)	0.013^†^	13 (31.7%)	7 (33.3%)	0.558
*0706	4 (6.5%)	35 (61.4%)	0.000^†^	2 (4.9%)	2 (9.5%)	0.417
*0707	20 (32.3%)	36 (63.2%)	0.013^†^	13 (31.7%)	7 (33.3%)	0.558
*0708	6 (9.7%)	35 (61.4%)	0.000^†^	3 (7.3%)	3 (14.3%)	0.325
*0709	20 (32.3%)	36 (63.2%)	0.013^†^	13 (31.7%)	7 (33.3%)	0.558
*0710N	20 (32.3%)	36 (63.2%)	0.013^†^	13 (31.7%)	7 (33.3%)	0.558
*0711	20 (32.3%)	36 (63.2%)	0.013^†^	13 (31.7%)	7 (33.3%)	0.558
*0712	4 (6.5%)	35 (61.4%)	0.000^†^	2 (4.9%)	2 (9.5%)	0.417
*0713	4 (6.5%)	35 (61.4%)	0.000^†^	2 (4.9%)	2 (9.5%)	0.417
*0714	6 (9.7%)	35 (61.4%)	0.000^†^	3 (7.3%)	3 (14.3%)	0.325
*08	4 (6.5%)	0 (0.0%)	0.570	3 (7.3%)	1 (4.8%)	1.000
*0801	2 (3.2%)	0 (0.0%)	0.434	1 (2.4%)	1 (4.8%)	0.661
*0816	2 (3.2%)	0 (0.0%)	0.434	1 (2.4%)	1 (4.8%)	0.566
*0833	1 (1.6%)	0 (0.0%)	1.000	1 (2.4%)	0 (0.0%)	0.661
*09	1 (1.6%)	0 (0.0%)	1.000	1 (2.4%)	0 (0.0%)	1.000
*0904	1 (1.6%)	0 (0.0%)	1.000	1 (2.4%)	0 (0.0%)	0.661
*0906	1 (1.6%)	0 (0.0%)	1.000	1 (2.4%)	0 (0.0%)	0.661
*10	3 (4.8%)	0 (0.0%)	0.282	3 (7.3%)	0 (0.0%)	0.545
*1001	3 (4.8%)	0 (0.0%)	0.282	3 (7.3%)	0 (0.0%)	0.282
*11	12 (19.4%)	7 (12.3%)	0.725	9 (22%)	3 (14.3%)	0.735
*1101	9 (14.5%)	22 (38.6%)	0.086	5 (12.2%)	4 (19%)	0.356
*1105	4 (6.5%)	8 (14%)	0.088	3 (7.3%)	1 (4.8%)	0.583
*1115	8 (12.9%)	3 (5.3%)	0.435	5 (12.2%)	3 (14.3%)	0.553
*1132	3 (4.8%)	3 (5.3%)	0.868	3 (7.3%)	0 (0.0%)	0.282
*1144	2 (3.2%)	6 (10.5)	0.358	1 (2.4%)	1 (4.8%)	0.566
*1166	1 (1.6%)	2 (3.5%)	0.586	0 (0.0%)	1 (4.8%)	0.339
*13	22 (35.5%)	3 (5.3%)	0.016^†^	19 (46.3%)	3 (14.3%)	0.014^#^
*1301	7 (11.3%)	12 (21.1%)	0.219	7 (17.1%)	0 (0%)	0.046^#^
*1303	7 (11.3%)	5 (8.8%)	0.525	5 (12.2%)	2 (9.5%)	0.558
*1361	7 (11.3%)	16 (28.1%)	0.079	7 (17.1%)	0 (0%)	0.046^#^
*1369	7 (11.3%)	3 (5.3%)	0.325	7 (17.1%)	0 (0%)	0.046^#^
*14	3 (4.8%)	0 (0.0%)	0.282	2 (4.9%)	1 (4.8%)	1.000
*1401	1 (1.6%)	0 (0.0%)	1.000	0 (0.0%)	1 (4.8%)	0.339
*1475	1 (1.6%)	0 (0.0%)	1.000	0 (0.0%)	1 (4.8%)	0.339
*15	11 (17.7%)	7 (12.3%)	0.722	10 (24.4%)	1 (4.8%)	0.048^#^
*1514	6 (9.7%)	3 (5.3%)	0.476	5 (12.2%)	1 (4.8%)	0.329
*1522	2 (3.2%)	9 (15.8%)	0.215	2 (4.9%)	0 (0.0%)	0.434

^#^ Significant difference at PC ≤ 0.05 between responder and nonresponder patients to treatment. ^†^Significant difference at PC ≤ 0.05 (corrected *P* after the Bonferroni correction) between patients and healthy group. Each patient may have multiple suballeles or none at all, so that totals are not always equal to “*n*.”

**Table 4:** Frequencies and percentages of linkage between HLA familial alleles in HCV patients and healthy group and in responder and nonresponder patients to combined treatment of interferon and ribavirin.

Haplotypes	HCV patients (*n* = 62)	Healthy controls (*n* = 57)	*P* value	Responders *N* = 41	Nonresponders *N* = 21	*P* value
DQB1*02-DQB1*03	15 (24.2%)	32 (56.1%)	0.015^†^	9 (23.7%)	6 (30%)	0.754
DQB1*02-DQB1*05	3 (4.8%)	10 (17.5%)	0.325	2 (5.3%)	1 (5%)	1.000
DQB1*02-DQB1*06	7 (11.3%)	4 (7%)	0.724	5 (13.2%)	2 (10%)	1.000
DQB1*03-DQB1*05	5 (8.1%)	10 (17.5%)	0.823	4 (10.5%)	1 (5%)	0.650
DQB1*03-DQB1*06	9 (14.5%)	3 (5.3%)	0.212	8 (21.1%)	1 (5%)	0.143
DQB1*05-DQB1*06	2 (3.2%)	4 (7%)	0.417	1 (2.6%)	1 (5%)	1.000
DQB1*02-DRB1*03	11 (17.7%)	2 (3.5%)	0.187	7 (18.4%)	4 (20%)	1.000
DQB1*02-DRB1*07	15 (24.2%)	35 (61.4%)	0.008^†^	10 (26.3%)	5 (25%)	1.000
DQB1*02-DRB1*13	7 (11.3%)	2 (3.5%)	0.222	6 (15.8%)	1 (5%)	0.403
DQB1*03-DRB1*03	10 (16.2%)	3 (5.3%)	0.250	6 (15.8%)	4 (20%)	0.724
DQB1*03-DRB1*04	11 (17.7%)	12 (21.1%)	0.219	5 (13.2%)	6 (30%)	0.163
DQB1*03-DRB1*07	10 (16.2%)	36 (63.2%)	0.000^†^	6 (15.8%)	4 (20%)	0.724
DQB1*03-DRB1*13	13 (21%)	1 (1.8%)	0.147	11 (28.9%)	2 (10%)	0.184
DQB1*05-DRB1*01	2 (3.2%)	6 (10.5%)	0.428	2 (5.3%)	0 (0%)	0.540
DQB1*05-DRB1*10	3 (4.8%)	0 (0.0%)	1.000	3 (7.9%)	0 (0%)	0.544
DQB1*06-DRB1*15	9 (14.5%)	4 (7%)	0.324	6 (15.8%)	3 (15%)	1.000
DRB1*03-DRB1*13	2 (3.2%)	3 (5.3%)	0.173	2 (4.9%)	0 (0%)	0.545
DRB1*04-DRB1*11	3 (4.8%)	7 (12.3%)	0.762	0 (0.0%)	3 (14.3%)	0.035^#^
DRB1*07-DRB1*13	4 (6.5%)	2 (3.5%)	0.071	3 (7.9%)	1 (4.8%)	1.000
DRB1*07-DRB1*15	3 (4.8%)	6 (10.5%)	0.421	1 (2.4%)	2 (9.5%)	0.263
DRB1*13-DRB1*14	2 (3.2%)	0 (0.0%)	0.568	0 (0%)	2 (9.5%)	0.111
DRB1*13-DRB1*15	2 (3.2%)	2 (3.5%)	0.247	2 (4.9%)	0 (0%)	0.545

^#^ Significant difference at PC ≤ 0.05 (corrected *P* after the Bonferroni correction) between responder and nonresponder patients to treatment. ^†^Significant difference at PC ≤ 0.05 (corrected *P* after the Bonferroni correction) between patients and healthy group. Each patient may have multiple suballeles or none at all, so that totals are not always equal to “*n*.”

**Table 5:** Frequencies and percentages of linkage between HLA alleles (haplotypes) in HCV patients and healthy group and in responder and nonresponder patients to combined treatment of interferon and ribavirin.

Haplotypes	HCV Patients (*n* = 62)	Healthy controls (*n* = 57)	PC	Responders *N* = 41	NonResponders *N* = 21	PC
DQB1*0201-DQB1*0202	28 (45.2%)	20 (35.1%)	0.078	18 (47.4%)	10 (50%)	0.534
DQB1*0202-DQB1*0205	7 (11.3%)	10 (17.5%)	0.695	6 (15.8%)	1 (5%)	0.226
DQB1*0204-DQB1*0301	14 (22.6%)	12 (21.1%)	1.000	9 (23.7%)	5 (25%)	0.577
DQB1*0204-DQB1*0313	3 (4.8%)	8 (14.1%)	0.567	0 (0%)	3 (15 %)	0.037^#^
DQB1*0309-DQB1*0313	7 (8.1%)	6 (10.5%)	1.000	2 (5.3%)	5 (25%)	0.041^#^
DQB1*0309-DQB1*0319	25 (40.3%)	18 (31.6%)	0.085	16 (42.1%)	9 (45%)	0.525
DQB1*0313-DQB1*0319	7 (11.3%)	6 (10.5%)	1.000	2 (5.3%)	5 (25%)	0.041^#^
DQB1*0603-DQB1*0628	10 (16.2%)	7 (12.3%)	0.447	8 (21.1%)	2 (10%)	0.250
DRB1*0301-DRB1*0318	8 (12.9%)	2 (3.5%)	0.087	6 (14.6%)	2 (9.5%)	0.447
DRB1*0706-DRB1*0711	4 (6.5%)	11 (19.3%)	0.084	2 (4.9%)	2 (9.5%)	0.417
DRB1*1301-DRB1*1328	7 (11.3%)	10 (17.5%)	0.695	7 (17.1%)	0 (0%)	0.046^#^
DRB1*1301-DRB1*1361	7 (11.3%)	12 (21.1%)	0.219	7 (17.1%)	0 (0%)	0.046^#^
DRB1*1301-DRB1*1369	7 (11.3%)	15 (26.3%)	0.089	7 (17.1%)	0 (0%)	0.046^#^
DRB1*1328-DRB1*1361	7 (11.3%)	3 (5.3%)	0.325	7 (17.1%)	0 (0%)	0.046^#^
DRB1*1328-DRB1*1369	7 (11.3%)	9 (15.8%)	0.752	7 (17.1%)	0 (0%)	0.046^#^

^#^ Significant difference at PC ≤ 0.05; (corrected *P* after the Bonferroni correction) between responder and nonresponder patients to treatment. Each patient may have multiple suballeles or none at all, so that totals are not always equal to “*n*.”
